# Allergens of *Arachis hypogaea* and the effect of processing on their detection by ELISA

**DOI:** 10.3402/fnr.v60.28945

**Published:** 2016-02-29

**Authors:** Amjad Iqbal, Farooq Shah, Muhammad Hamayun, Ayaz Ahmad, Anwar Hussain, Muhammad Waqas, Sang-Mo Kang, In-Jung Lee

**Affiliations:** 1 Department of Food Safety and Food Quality, University of Gent, Gent, Belgium; 2 Department of Agriculture, Abdul Wali Khan University Mardan, Mardan, Pakistan; 3 Department of Botany, Abdul Wali Khan University Mardan, Mardan, Pakistan; 4 Department of Biotechnology, Abdul Wali Khan University Mardan, Mardan, Pakistan; 5 School of Applied Biosciences, College of Agriculture and Life Science, Kyungpook National University, Daegu, South Korea

**Keywords:** peanut proteins, anaphylaxis, conglutin, vicilin, glycinin, allergens processing

## Abstract

Food allergies are an emerging public health problem in industrialized areas of the world. They represent a considerable health problem in these areas because of the relatively high number of reported cases. Usually, food allergens are proteins or glycoproteins with a molecular mass ranging from 10 to 70 kDa. Among the food allergies, peanut is accounted to be responsible for more than 50% of the food allergy fatalities. Threshold doses for peanut allergenic reactions have been found to range from as low as 100 µg to 1 g of peanut protein, which equal to 400 µg to 4 g peanut meal. Allergens from peanut are mainly seed storage proteins that are composed of conglutin, vicilin, and glycinin families. Several peanut proteins have been identified to induce allergic reactions, particularly Ara h 1–11. This review is mainly focused on different classes of peanut allergens, the effect of thermal and chemical treatment of peanut allergens on the IgY binding and detectability of these allergens by enzyme linked immunosorbent assay (ELISA) to provide knowledge for food industry.

Food allergies are becoming a main public health concern in well-established areas, which represents a significant threat to human health in recent years (1, 2). The occurrence of food allergies has amplified over the years, which has confronted both allergologists and food scientists with serious challenges. The term ‘food allergy’ has been defined by the European Academy of Allergology and Clinical Immunology (EAACI) as ‘… a hypersensitivity reaction initiated by immunological mechanisms’. Food allergens are actually the constituents in foods that aggravate immunological reactions. The adverse/hypersensitive reactions to foods might be poisonous or non-poisonous reactions; the latter might be immune-mediated or non-immune-mediated opposing reactions. The food's adverse effect that involves the immune system reaction is an absolutely IgE-mediated allergy (3). Generally, the most common allergenic food worldwide includes peanut, tree nuts, milk, soybean, crustacean, egg, fish, and wheat. Among the offending food, peanuts are the main culprits that cause severe allergies and even life-threatening anaphylactic shocks (4).

The cultivation of peanut or groundnut (*Arachis hypogaea* L.), began in Bolivia, South America, but today it is grown throughout various ecological zones of the world. The crop was initially grown by the natives during European expansion in the sixteenth century and was thereafter grown in Europe, Africa, Asia, and the Pacific Islands. Peanut belongs to the family Fabaceae or legumes and is the world's third important oil and food crop, following soybean and cotton. Asian countries, including India and China, and the United States have been the leading producers for more than 25 years and provide 70% of the world's peanuts. Peanuts are grown mainly for human consumption in the form of whole seed or processed to produce butter, cooking oil, and various other products.

Beside all these benefits, peanuts have some proteins known as peanut allergens. These allergens are of particular interest because of the reported life-threatening consequences of anaphylactic shock. Peanut allergy causes significant health-care problems globally and currently affects 0.6–1.5% of the children in developed countries (5). The prevalence is, however, different in different parts of the world and is increasing in developed nations (6). There is less peanut allergy in China than in the United States, despite similar levels of peanut consumption (7), possibly because of differences in food preparation. For example, dry roasted peanuts are more allergic than boiled or fried peanuts. Nevertheless, it remains unclear why the peanut allergy prevalence is lower in China than in Europe and America (8–12).

Presently, peanut allergy diagnostic includes double-blind, placebo-controlled food challenge (DBPCFC), the specific skin prick test (SPT), the basophil activation test, and the measurement of specific IgE (13, 14). The major reason in the failure of these tests is the existence of the ‘peanut-sensitized’ individuals. Those individuals, despite of having peanut-specific IgE antibodies, consume peanuts without any symptoms. Among a group of 8-year-old children, only 10% had peanut-specific IgE antibodies, but only approximately 2% were truly allergic (15).

Nowadays, the ELISA technique is the most commonly used immunoassay in the laboratories of the food industry to detect and quantify hidden allergens in food. ELISA has been successfully used over the years as a preferred method to detect allergens in meat and meat products, fish and fish products, milk and milk products, soyabean, nuts and nut-based products, and fruit juices and ingredients (16, 17). The method has the advantages of high sensitivity, low cost, fast application, ease of use, reliability, and speed. With ELISA tests, allergens or specific marker proteins can be detected by colorimetric reaction after the binding of the antigen with a specific enzyme-labeled antibody.

## Composition of peanut proteins

The presence of the various proteins in the tested peanut samples from different parts of the world was found same, but the amounts were different. Beside this, the IgE-binding properties of different peanut varieties were also the same as Koppelman et al. (18). Highly processed oil (acid extracted, heat distilled), on the other hand, does not contain peanut protein and can safely be consumed by allergic patients. However, the cold-pressed or cold-extruded peanut oils, with processing at lower temperatures, may contain traces of peanut protein and may induce allergic reactions in allergic subjects (19).

## Allergens in peanut

The seeds of peanut contain over 32 different proteins (20) but only 18 have been reported to have an allergen property and 11 have been identified (21). Among the various isolated allergens from peanut (Ara h 1–11), Ara h 2 and Ara h 6 are the most important with regard to food allergy (22). Indeed, peanut allergens, in particular, are more important than other food allergens because they have shown to be extremely resistant to digestion, denaturation from heat, acidity, alkali, and proteolytic activities (23, 24). Allergens that have been isolated include cupin (vicilin-type, 7S globulin), conglutin (2S albumin), cupin (legumin-type, 11S globulin, glycinin), cupin (legumin-type, 11S, glycinin), profilin, pathogenesis-related protein, PR-10, nonspecific lipid-transfer protein 1, 14 kDa oleosin, and 16 kDa oleosin (20). Lipid-transfer proteins (LTPs) are heat stable and resistant to proteolytic digestion (25) and can cross-react with a broad range of food allergens (26). Due to the extreme resistance of LTPs to pepsin digestion, LTPs in particular are potentially severe food allergens (27).

## Properties of peanut allergens

### Ara h 1

Ara h 1 or conarachin belongs to the vicilin family, a seed storage proteins (globulins). The molecular weight of this glycoprotein is about 65,000. This protein is similar to the conglycinin from soy proteins with the major IgE epitopes within this extension region. Ara h 1 is a protein with high thermal stability but showed minor structural changes in 5M urea. It has also been observed that few of the IgE-binding epitopes of Ara h1 are resistant to pepsin degradation (28).

### Ara h 2

Ara h 2 is a glycoprotein of 17.5 kDa and was initially identified from crude peanut extracts. Ara h 2, a glycoprotein with an isoelectric point (pI) of 5.2 that resembles to a protein from 2S albumin family, that is, delta conglutin. Ara h 2 is known to be a storage protein that can act as a trypsin inhibitor (25). The Ara h 2 is an aciduric protein that can withstand degradation by digestive enzymes, which might be why it is recognized by serum IgE from most peanut-allergic patients (29).

### Ara h 3

Peanut is one of the well-known sources of allergens, and among those allergens, Ara h 3 is the major and complex one (30). Ara h 3 is a single-chain polypeptide of 60 kDa and belongs to 11S storage protein from the glycinin family with a less stable response to the enzymatic (pepsin) action than the Ara h 2 and Ara h 6 (31). The extensively proteolytically processed protein is bound by disulphide bridges and is found in trimeric and hexameric structures. This oligomeric structure limits the determination of allerginisity of the Ara h 3, as there is no specific IgE available but the polyclonal antibody raised against the oligomers will solve this problem (32).

### Ara h 4

Ara h 4 has 35.9 kDa acidic subunit with a pI of 5.5, and the amino acid sequences of both Ara h 3 and Ara h 4 are 91% identical and considered isoallergens (33).

### Ara h 5

Profilinin or Ara h 5 is a 12 to 15 kDa monomeric actin-binding protein present in all eukaryotic cells. It was reported to be a minor allergen in birch pollen but is now considered a ubiquitous panallergen found in peanuts, hazelnuts, pear, tomato, and so on. Profiling has been involved in the birch-mugwort-celery-spice syndrome, and several studies concluded that this protein can also play role in patients allergic to hazelnut, celery, carrot, peanuts, peach, pear, apple, potato, tomato, and pumpkin seed. However, recent studies suggested that profilin sensitization has little or no clinical relevance (34).

### Ara h 6

Ara h 6, a 15 kDa allergen, has been isolated, and is recognized by 20 out of 29 peanut-allergic patients on IgE-immunoblot. The potent biological functionality of Ara h 6 is demonstrated by the degranulation of basophils, even at concentrations below 10 pg/mL, and by positive skin prick reactions. Ara h 6 has homology to Ara h 2, especially in the middle part and at the C-terminal part of the protein. This demonstrates that at least part of the epitopes of Ara h 6 is cross-reactive with epitopes on Ara h 2. The potent biological functionality of Ara h 6 is demonstrated by the release of histamine from basophils; even at concentrations below 10 µg/ml (35).

### Ara h 7

Another storage protein from conglutin family is an Ara h 7 with 15.8 kDa *Mw* and pI of 5.6. The amino acid sequence of Ara h 7 is 53% similar to Ara h 6, while 35% similar to Ara h 2 (33), but are less stable than both due to conservation of only two disulphide bonding. Schmidt et al., (36) enriched and separated peanut proteins of molecular weight less than 20 kDa on 2D gel electrophoresis. After mass spectroscopic analysis, two isoallergens were found, one of which had an additional pro-peptide cleavage point. Furthermore, the putative cleavage point demonstrates the biological function of conglutins as an amylase/trypsin inhibitor.

### Ara h 8

Unlike Ara h 1–6, which are seed storage proteins, Ara h 8 belongs to pathogenesis-related protein family PR–10. Ara h 8 is also heat labile and prone to proteolytic digestion. This allergen is homologous with birch pollen allergen and because of its cross-reactivity, it is very important for birch pollen allergic patients (33, 37, 38).

### Ara h 9

Ara h 9 is a nonspecific lipid-transfer protein 1, which was indentified clearly in 2009 (39). There two isoforms of Ara h 9 have been established, and the amino acid sequences of both isoforms are 90% identical. Beside similar IgE reactivity, both also have 60–70% identical amino acid sequence with LTPs from other food (e.g. hazelnut, chestnut, almond, peach, pear, plum, cherry, strawberry, lentils, lupin, sunflower, beans, pea) (40, 41).

### Ara h 10 and Ara h 11

Both types of peanut allergens belong to the oleosin family. Ara h 10 is approximately 16 kDa, while Ara h 11 is 14 kD. Both can be obtained from oil bodies of peanut and need to be studied in depth (20).

## Sensitization to peanut proteins

**Fig. 1:**
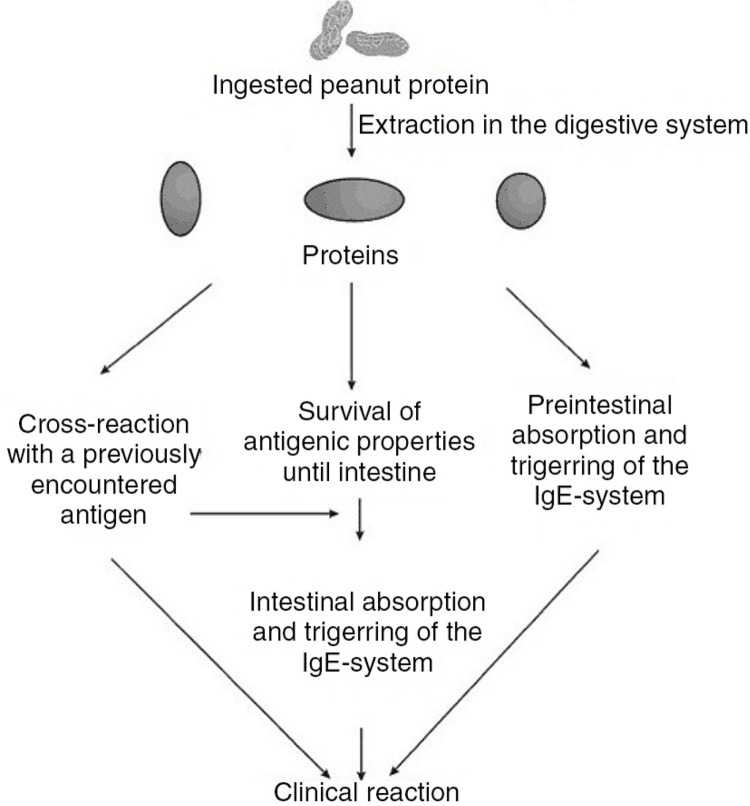
Sequence leading to sensitization and elicitation of a food allergic reaction to a food.

Peanut allergy usually presents after a period of sensitization to peanut. However, some children have severe reactions as a result of their apparent first exposure to some allergens through breast milk (42). Several studies have documented an epidemiologic relationship between the increased consumption of peanut by pregnant and breast-feeding mothers and the likelihood of the allergic sensitization of their children. These studies suggest a transfer of maternal dietary peanut protein to breast milk may predispose at-risk children to occult sensitization. Since sensitization requires prior exposure to generate allergen-specific IgE, the sensitizing exposure must be occult, in many cases. According to Vadas et al., (43), peanut proteins appeared with 1 to 3 h following oral ingestion. Both low- and high-molecular weight proteins with mobilities corresponding to Ara h 1 and Ara h 2 were secreted intact into breast milk with no evidence of degradation (18). The mechanism of action of sensitization was given by Poulsen (44) (Fig. 1).

## Effect of processing on the detection of allergens

For many foods, thermal processing is necessary and unavoidable and may include drying, baking, frying, microwave treatment, roasting, frying, or boiling. It is often thought that thermal processing may affect the structure of the protein, which in turn influences the detection of the proteins or allergens. The extent of the physicochemical impact on protein structure and functionality depends largely on the intrinsic characteristics of protein, the temperature applied, the duration of heat treatment, and the pH. The loss of tertiary structure can create new epitopes, e.g. by unfolding and exposing the formerly hidden sites, as well as destroying the existing sites (29, 45). Typically, the loss of secondary structure occurs at a temperature between 55°C and 70°C, the cleavage of disulfide bonds occurs at 70°C−80°C, the formation of new intra/intermolecular interactions and rearrangements of the disulfide bonds at 80°C−90°C, and the formation of aggregates at 90°C−100°C. Besides those physical transformations, the chemical modification of protein may also occur at high temperatures, such as 100°C−125°C. One of the most important reactions is that of protein amino groups with sugars, leading to an impressive cocktail of advance glycation end products, such as Millard reaction products. Thermal processing will also reduce the solubility of target protein, which can reduce the extractability of soluble proteins, the basis for the detectability of allergenic food constituents in food products. Roasted peanuts, for instance, are widely used for food businesses to enhance the flavor of the raw ingredient, yet the allergenic protein is less soluble in the aqueous solutions required for detection (8). In addition, antigen recognition by immunological detection is adversely affected by processing because it can denature, alter, or remove proteins so that they are no longer detectable by the antibodies used in the assays. Millard reaction products have been shown to have an inhibitory effect on IgE binding of proteins (46).

## Effect of heat treatment of peanut proteins on IgY binding

**Fig. 2:**
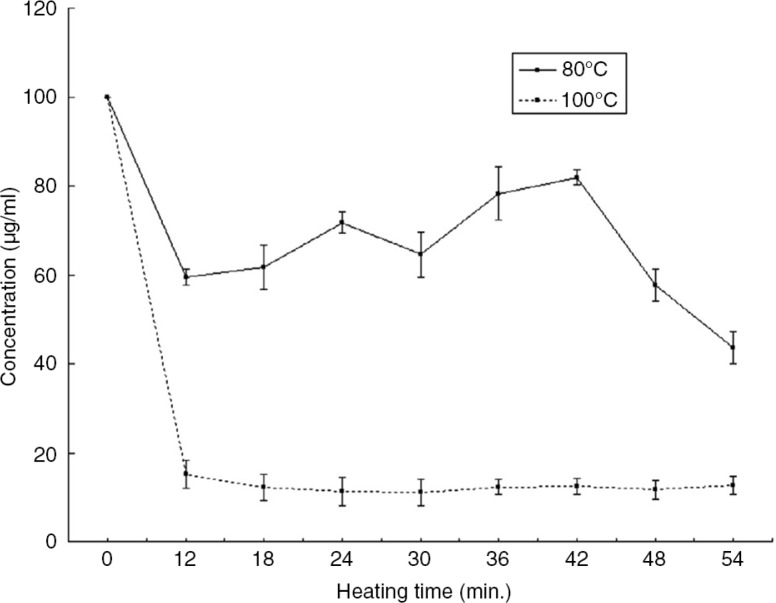
Detectability of peanut proteins after heat treatment at 80°C and 100°C in PBS (24).

Iqbal and Ateeq (24) observed the effect of heat treatment on the detectability of peanut proteins by Chicken IgYs (Fig. 2). The concentration of peanut proteins in PBS before heat treatment shows a good sensitivity, but after heat treatment there was a low detection of peanut concentration, which could be correlated to the loss of IgY binding due to conformational changes of peanut proteins. After certain period of time, the protein concentration became constant because of heat stable fractions, which is an indication that certain fractions of epitopes were not affected by heat and hence retained their IgY binding capacity (24, 47).

## Effect of pH on the detectability of peanut proteins

**Fig. 3:**
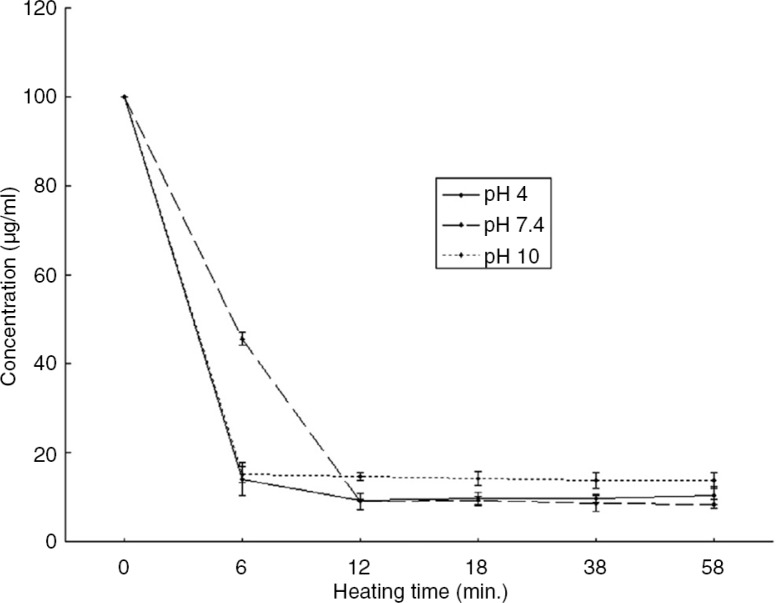
Detectability of peanut protein after heat treatment at 100°C in PBS buffer at pH 4, pH 7.4 and pH 10 (24).

Heating peanut antigens at 100°C in normal, acidic, and alkali conditions has an effect on the IgY binding. This might be due to the conformational changes of the protein molecules because both antibodies and antigens are proteins and can be affected by the changes in pH. The alkaline and acidic conditions have almost the same negative effect on the binding of IgY, but the severity in alkaline conditions can be high (Fig. 3). The denaturation of proteins in alkaline conditions might be marginally faster because of the hydrolysis of proteins that occurs faster in alkaline conditions than in acidic conditions (24).

## Effect of reducing sugar of peanut proteins on IgY binding

**Fig. 4:**
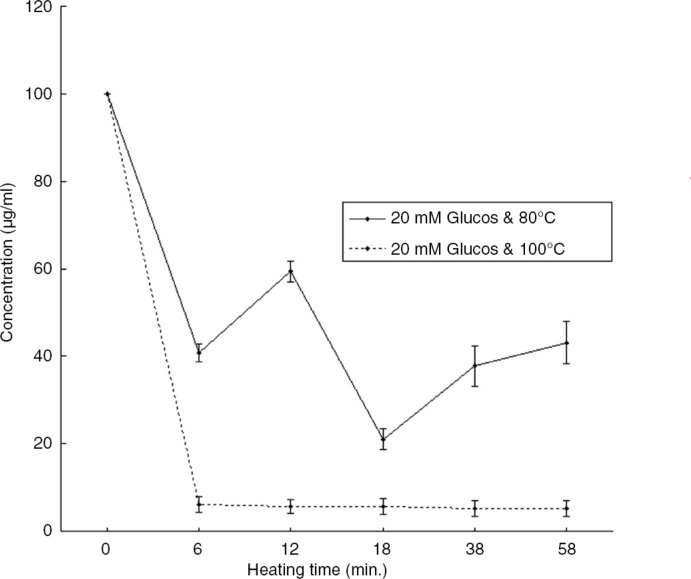
Detectability of peanut protein after heat treatment at 80°C in 20 mM glucose solution and 100°C in 20 mM glucose solution (24).

The effect of heating in 20 mM glucose at 100°C can results in a Maillard reaction between an amino acid (peanut proteins) and a reducing sugar (glucose). The Maillard products may interfere with the IgY binding by altering the protein resulting in the antibody not being able to recognize the antigen protein anymore (Fig. 4) (24).

## Conclusion

Many foods require thermal processing from an aromatic and microbial point of view, which includes drying, baking, frying, microwave treatment, roasting, frying, or boiling. It is possible that such processing may affect the structure of the protein, which in turn influences the detection of these proteins or allergens. The extent of physicochemical impact on protein structure and functionality depends largely on the intrinsic characteristics of proteins, the temperature applied, the duration of heat treatment, and the pH. Thermal processing may reduce the solubility of the target protein, which is the basis for the detectability of allergenic food constituents in food products. Certainly peanuts are the most common of the allergens and are a good source of allergenic proteins. Roasting these proteins (allergens) might change the confirmation of the native protein to a protein with high allergenicity and low detection. Therefore, a cheap, robust, and reliable biochemical method is needed to protect the consumer from life-threatening allergens. Enzyme linked immunosorbent assay is one of the best options to overcome these hurdles. The method is not as sensitive as polymerase chain reaction, but is still cheap, offers high throughput, and is reliable enough to detect altered allergenic protein fractions. Also, different processing conditions can completely change the protein's immunochemical characteristics; the risk of masking the antigen is high. This is much more likely to happen when using monoclonal antibodies, which are specific for a particular protein, but using polyclonal antibodies, reduces this risk.
